# Assessing effectiveness of *Komagataeibacter* strains for producing surface-microstructured cellulose via guided assembly-based biolithography

**DOI:** 10.1038/s41598-021-98705-2

**Published:** 2021-09-29

**Authors:** Marcello Brugnoli, Francesco Robotti, Salvatore La China, Kavitha Anguluri, Hossein Haghighi, Simone Bottan, Aldo Ferrari, Maria Gullo

**Affiliations:** 1 Department of Life Sciences, University of Modena and Reggio Emilia, 42123 Reggio Emilia, Italy; 2 Hylomorph AG, Zurich, Switzerland

**Keywords:** Biomedical engineering, Applied microbiology, Biotechnology, Microbiology

## Abstract

In this study, a medical device made of surface microstructured bacterial cellulose was produced using cellulose-producing acetic acid bacteria wild-type strains in combination with guided assembly-based biolithography. The medical device aims at interfering with the cell's focal adhesion establishment and maturation around implantable devices placed in soft tissues by the symmetrical array on its surface. A total of 25 *Komagataeibacter* strains was evaluated over a three-step selection. In the first step, the ability of strains to produce a suitable bacterial cellulose layer with high production yield was examined, then nine strains, with a uniform and smooth layer of bacterial cellulose, were cultured in a custom-made silicone bioreactor and finally the characteristics of the symmetrical array of topographic features on the surface were analysed. Selected strains showed high inter and intra species variability in bacterial cellulose production. The devices obtained by K2G30, K1G4, DSM 46590 (*Komagataeibacter xylinus*), K2A8 (*Komagataeibacter sp.*) and DSM 15973^T^ (*Komagataeibacter sucrofermentas)* strains were pouched-formed with hexagonal surface pattern required for reducing the formation of fibrotic tissue around devices, once they are implanted in soft tissues. Our findings revealed the effectiveness of the selected *Komagataeibacter* wild-type strains in producing surface microstructured bacterial cellulose pouches for making biomedical devices.

## Introduction

The search for biopolymers with innovative attributes is a challenge for the biotechnological industry. In this frame, bacterial cellulose (BC) in the native and functionalized form has received extensive attention due to features, such as high-water holding capacity, high light transparency, non-toxicity, purity and biocompatibility^1,2^. Based on the structural and safety characteristics of BC and its derivates, this biopolymer has been proposed in several fields such as food, textile, pharmaceutical, biomedical, cosmetic, environmental and engineering applications^3–9^. According to the United States Food and Drug Administration (FDA), BC is is “generally recognized as safe” (GRAS)^10,11^. Recently, the European Food Safety Authority (EFSA) Scientific Panel on Biological Hazards (BIOHAZ) included the species *Komagataeibacter sucrofermentans* in the list of QPS-recommended biological agents, intentionally added to food^12^. Previous studies highlighted the absence of BC cytotoxicity on mouse fibroblast cells and its suitability as the carrier of active medical and cosmetic formulations^13,14^.

For instance, BC could be used as a vehicle for antibiotics or medicines, allowing their transfer to the wound. At the same time, it acts as a physical barrier against external infections^3,15^. Moreover, BC is adaptable to the wound surface and provides an exudate absorption thus it is possible to use it as a matrix for the epithelialization of burns even of third-degree^15,16^. BC grafts might potentially reduce the rejection rates of transplanted corneas and improve the treatment of eye diseases (e.g., age-related macular degeneration) mainly due to the augmenting local neovascularization, diminishing side effects, and surgical recovery intervals^17^. However, it is widely known that the human body recognizes the foreign material immediately after implantation, and it could trigger an inflammatory response followed by a sequence of events that lead to the deposition of fibrotic tissue^18,19^. Such an event is correlated with several health risks for patients, both during control interventions and after, when the functionality of the implanted device is required^20^.

The intrinsic characteristics of BC, such as the non-toxic chemical composition, the purity, the high porosity, the bulk mechanical properties, and the matrix-like morphology, make it advantageous in biomedical applications to prevent the fibrotic adhesion, as documented by several studies^15,21–23^. Indeed, it was previously reported that the adverse conditions for adhesion, between cells and BC, are established by an isotropic distribution of topographical features, which physically interfere with the establishment and maturation of focal adhesion^21,24^. The micro-structuration of BC’s surface is possible using a Guided Assembly-Based Biolithography (GAB), which is a powerful replica molding methodology to transfer on-demand functional topographies to the surface of BC nanofiber textures. BC nanofibers are assembled in a three-dimensional network reproducing the hexagonal pattern imposed by the mold^20,24,25^. Thus, the formation of the pattern on the surface of BC inhibits the initial biological recognition^22^ avoiding the formation of fibrotic tissue around implantable devices placed in soft tissues.

Among BC producing organisms, acetic acid bacteria (AAB) of the *Komagataeibacter* genus comprise species such as *Komagataeibacter xylinus, K. hansenii and K. sucrofermentas* with high production yield which are reported as native or functionalized biomaterial pruducers^1^.

In the present work, 25 AAB wild-type strains belonging to the *Komagataeibacter* genus were evaluated for their suitability to synthesize a medical device made of surface microstructured BC in combination with GAB.

Due to high intraspecific variability within AAB in producing BC^26^, 9 strains were selected based on BC production yield and shape. Selected strains were cultured inside the bioreactor and their characteristics of the symmetrical array on the surface were analysed. Promising outcomes have been obtained by 5 strains, which produced the required surface-microstructured BC. This study provides new evidence for the use of wild-type AAB strains as biological machineries for producing biomedical devices.

## Results and discussion

### Assessment of bacterial cellulose production by *Komagataeibacter* strains

The amount and shape of BC produced by AAB strains are presented in Table 1. AAB species considered for this study were *K. xylinus*, *K. hansenii*, and *K. sucrofermentans*, which are reported as the highest BC producers among the species of the *Komagataeibacter* genus^27–32^. BC was produced by all the strains; however, a great variability was observed not only on the macroscopic structure of the native BC (Supplementary Fig. S1), but also in terms of weight. Some strains produced a uniform and homogenous BC layer, smooth on the surface, which was easily removed from the culture broth without damaging its shape. Whereas other strains produced a fragmented BC layer with a heterogenous and compact macrostructure. Once removed from the culture broth the cellulosic matrix has lost its original shape.

**Table 1:** Weight of dried BC and shape of native BC produced by AAB strains used in this study and their isolation source. Values are given as mean ± standard deviation (n = 3).

Strain	Dried BC (g)	Native BC in liquid cultivation	Isolation source	Species
K1A18^26^	0.0168^n^ ± 0.0001	Uniform and smooth	Liquid kombucha tea fraction	*Komagataeibacter *sp.
**K1G4 = UMCC2947**^31^	0.2096^a^ ± 0.0001	Uniform and smooth	Liquid kombucha tea fraction	*K. xylinus*
**K1G23**^26^	0.0629^b^ ± 0.0001	Uniform and smooth	Liquid kombucha tea fraction	*Komagataeibacter *sp.
**K2A8**^26^	0.0401^e^ ± 0.0001	Uniform and smooth	Liquid kombucha tea fraction	*Komagataeibacter *sp.
K2A10 = UMCC 2965^26^	0.0162^n^ ± 0.0001	Uniform	Liquid kombucha tea fraction	*Komagataeibacter *sp.
K2A8^26^	0.0293^g^ ± 0.0002	Uniform and smooth	Liquid kombucha tea fraction	*Komagataeibacter *sp.
K2G8^26^	0.0296^g^ ± 0.0001	Uniform and smooth	Liquid kombucha tea fraction	*Komagataeibacter *sp.
K2G10^26^	0.0230^l^ ± 0.0001	Uniform	Liquid kombucha tea fraction	*Komagataeibacter *sp.
K2G14^26^	0.0317^f^ ± 0.0001	Uniform	Liquid kombucha tea fraction	*Komagataeibacter *sp.
K2G15^26^	0.0264^i^ ± 0.0001	Uniform	Liquid kombucha tea fraction	*Komagataeibacter *sp.
**K2G30 = UMCC2756**^33^	0.0519^d^ ± 0.0002	Uniform and smooth	Pellicle kombucha tea fraction	*K. xylinus*
**K2G39 = UMCC2970**^26^	0.0540^c^ ± 0.0002	Uniform and smooth	Liquid kombucha tea fraction	*Komagataeibacter *sp.
K2G41 = UMCC 2971^26^	0.0247^k^ ± 0.0001	Uniform and smooth	Liquid kombucha tea fraction	*Komagataeibacter *sp.
K2G44 = UMCC 2972^26^	0.0132^p^ ± 0.0001	Uniform	Pellicle kombucha tea fraction	*Komagataeibacter *sp.
**DSM15973**^**T**^^34^	0.0164^n^ ± 0.0001	Uniform and smooth	Black cherry	*K. sucrofermentas*
DSM 2004^35^	0.0294^g^ ± 0.0001	Uniform	Unknown source	*K. xylinus*
DSM 2325^35^	0.0276^h^ ± 0.0006	Uniform	Unknown source	*K. xylinus*
**DSM46590**	0.0255^j^ ± 0.0001	Uniform and smooth	Unknown source	*K. xylinus*
**DSM46591**	0.0194^m^ ± 0.0002	Uniform and smooth	Unknown source	*K. xylinus*
DSM 46602^36^	0.0064^q^ ± 0.0001	Fragmented	Vinegar	*K. xylinus*
DSM 46603^36^	0.0127^p^ ± 0.0001	Fragmented	Unknown source	*K. xylinus*
**DSM46604**^37^	0.0155° ± 0.0005	Uniform and smooth	Unknown source	*K. xylinus*
DSM 46605^36^	0.0041^r^ ± 0.0004	Fragmented	Vinegar brew	*K. xylinus*
DSM 6513^T^ ^37^	0.0047^r^ ± 0.0002	Fragmented	Mountain ash berries	*K. xylinus*
DSM 5602^T^ ^38^	ND	Fragmented	Vinegar	*K. hansenii*

Different lowercase letters in the same column indicate significant differences (p < 0.05).

Bold fonts refer to strains that were chosen for surface-microstructured BC production.

*ND* not detectable.

Considering this study aimed to synthesize a medical device made of surface-microstructured BC, 9 strains producing uniform and smooth BC layers of different weights (Supplementary Fig. S2) were selected for further investigation. Among them, 5 strains were isolated from Kombucha tea, one from black cherry and 3 from unknown isolation sources (Table 1).

The variability in BC production has been previously observed for strains of the genus *Komagataeibacter* and within strains of the same species (e.g., *K. xylinus*). In the *Komagataeibacter* genus, differences in cellulose synthase (CS) complex have been correlated to a different ability in BC production^39^. The main reason for this difference is due to the number of bcs operons, which generally span from 1 to 3 in members of the *Komagataeibacter* genus^39,40^. However in *K. xylinus* species two strains were described to possess a fourth copy of bcsAB gene^33,41^. We previously obtained and analysed K2G30 and K1G4 genomes. The K2G30 genome possesses three bcs operons and a fourth copy of bcsAB gene, that encodes the catalytic core of CS. Whereas K1G4 analysis revealed the presence of the two bcs operon types structurally completed and a third copy of bcsAB gene^31,33^. These features can explain the high amount of BC produced by these two strains.

Other factors that contribute to strain variability in BC production are, the isolation source and handling of culture. Most of the strains of this study originated from food matrices, especially Kombucha tea, which is considered a selective source for the recovery of BC producing AAB^21^, while others were originally collected from sugared and acidic products. The laboratory culturing of strains also affects the stability of phenotypic traits. This phenomenon is already observed for AAB when they are continuously cultivated and preserved by short-time preservation methods, which increase the formation of high rate of spontaneous mutants^42,43^.

### Production of surface-structured bacterial cellulose with guided assembly-based biolithography

**Figure 1:**
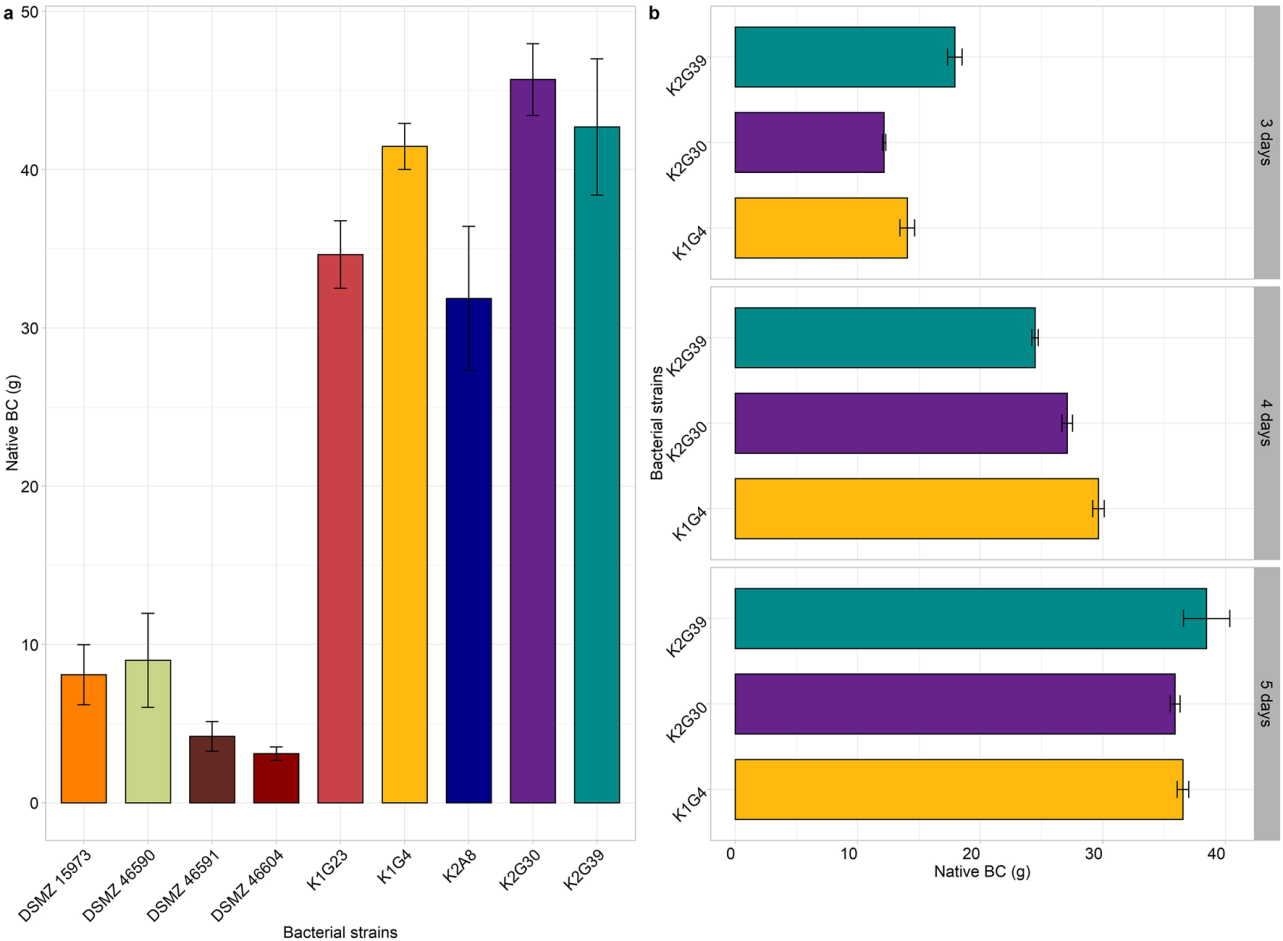
Weight of native BC produced by screened strains. BC weight was obtained after incubation at 28 °C for 7 **(a)** and 3, 4, 5 **(b)** days inside bioreactor. Values are given as mean ± standard deviation (n = 3).

Cultures derived from the nine selected strains were tested in the polydimethylsiloxane (PDMS) bioreactor, using the same conditions as during the previous tests (Hestrin–Schramm^44^ (HS) broth; 5% v/v inoculum; incubation at 28 °C for 7 days). Outcomes confirmed the great variability in native BC weight (Fig. 1a) (Supplementary Table S1) and characteristics. Some strains differed from the others to produce non-optimal BC pouches. K1G4, K2G30 and K2G39 strains produced the highest amount of BC, but the formed pouch did not have optimal attributes. Also, the visual analysis of transparency and thickness of the pouches varied among strains (Data not shown). To assess the suitability of the strains for the purpose of this study, it has been hypothesized to reduce the incubation time to 3, 4, and 5 days for the highest producers (K2G30, K2G39, and K1G4) (Fig. 1b). Results showed that the optimal production required 3 days of cultivation. The yield of BC production and its characteristics are also influenced by the type of vessel in which the microbial strains grow. Considering AAB strictly aerobic organisms, in the static cultivation regime, BC is formed at specific sites of the air surface of liquids^45^. Therefore, production in terms of yield is dependent on the ratio between the surface exposed to air and the volume (S/V ratio) of the vessel, in which bacteria grow.

The bioreactor used in this study allows the transfer of oxygen, which is a key factor in the production of BC by the *Komagataeibacter* sp. The constant diffusion of oxygen is permitted by the non-polar nature of the PDMS^46^. Indeed, the lower the polarity of a material is, the higher is the permeability to the oxygen, as reported for other materials, such as film composites with whey protein isolate and pectin^47,48^. Therefore, a higher BC yield was reached for most of the culture strains due to the bigger gas–liquid interface (Table 2). Moreover, oxygen inside the bioreactor was further increased by the high permeability of PDMS^49,50^.

**Table 2:** Dried BC yield (g/L) produced after incubation at 28 °C for 7 days inside flasks and bioreactor. Values are given as mean ± standard deviation (n = 3).

Strain	Flask (30 mL)	Bioreactor (55 mL)
	Yield (g/L) ± st dev (g/L)	Yield (g/L) ± st dev (g/L)
K1G4	6.9867^a^ ± 0.0033	3.7020^bc^ ± 0.4153
K1G23	2.0967^b^ ± 0.0033	2.8348^cd^ ± 0.2950
K2G39	1.8011^c^ ± 0.0069	3.8764^b^ ± 0.4344
K2G30	1.7311^d^ ± 0.0051	4.8939^a^ ± 0.2734
K2A8	1.3367^e^ ± 0.0033	2.5028^d^ ± 0.5575
DSM 15973^T^	0.5456^f^ ± 0.0019	0.8077^e^ ± 0.0387
DSM 46604	0.5156^g^ ± 0.0168	0.4907^e^ ± 0.0621
DSM 46590	0.8511^h^ ± 0.0019	0.8721^e^ ± 0.2112
DSM 46591	0.6478^i^ ± 0.0051	0.4992^e^ ± 0.0705

Different lowercase letters in the same column indicate significant differences (p < 0.05).

Considering the cultivation method used in this study, the gas–liquid interface area of the 100 mL flask was approximately 9.42 cm^2^, whereas in the case of the bioreactor it was nearly 120 cm^2^, of which just 9.5 cm^2^ of the free interface. Consequently, by approximating the geometric structure of the 100 mL flask to a truncated cone with diameters of 6.60 cm and 6.00 cm, and by approximating the structure of the bioreactor to a parallelepiped, the S/ V ratios were 0.60 cm^2^/cm^3^ and 1.75 cm^2^/cm^3^, respectively.

Even though the higher BC production occurred inside the PDMS bioreactors, the size of the pouches produced by strain DSM 46604 was smaller than that required, and it collapsed, not standing at the level at which the bacterial culture had been inoculated. Once the strain was grown inside the PDMS bioreactor, the structure of the pocket composed by BC, to support its sidewalls, has nothing but the BC itself. The collapse of the structure could be correlated to the slowness of the BC pace synthesis by the microbial strain, which leads to the absence of a unique and packed biofilm BC formation inside the bioreactor. Therefore, the DSM 46604 strain did not produce satisfactory results from a structural point of view.

**Figure 2:**
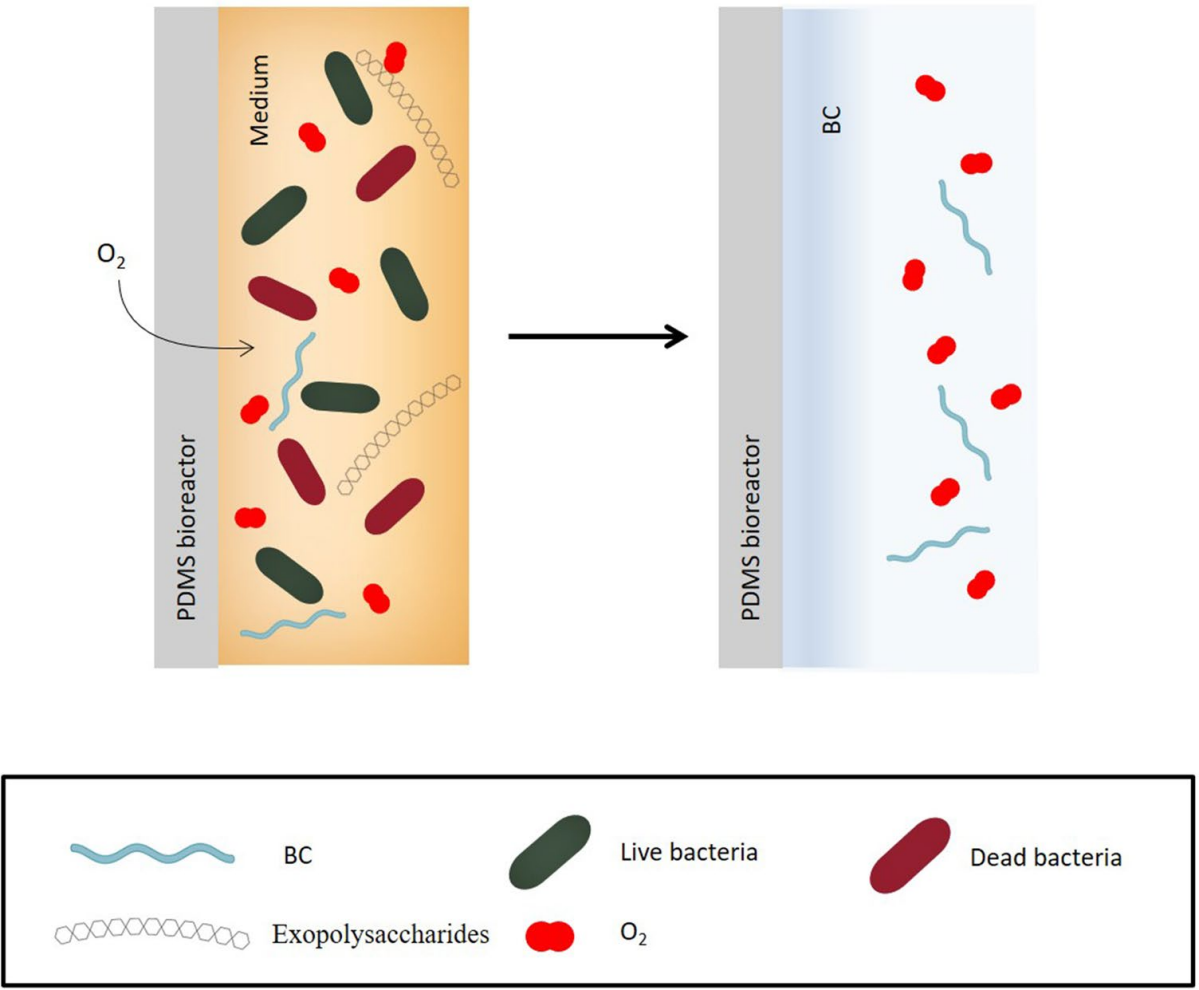
Formation of BC layer inside the PDMS bioreactor.

As already mentioned, through a continuous exchange of oxygen, bacteria can produce BC also within the walls of the bioreactor (Fig. 2), even though more slowly than the upper part, which is directly in contact with the air. Therefore, a thick layer of BC was observed for almost all strains at the open liquid–air interface at the end of incubation time. A sort of closed cap, sealing the top of the surface-microstructured BC pouch was formed. That cap was easily removed from the rest of the BC and the devices assumed the pouch-shape, without any damage to the BC. Most of tested strains formed the removable cap with two exceptions. In surface-microstructured BC produced by DSM 46591 and DSM 46604, the thicker layer was not visible and, consequently, not possible to remove without damaging the rest of the BC.

Our outcomes highlight that the use of defined AAB strains is a versatile strategy that allows obtaining costumed devices, modulating the growth conditions. Moreover, for some strains a reduction in cultivation time was proved, which is a further milestone for using AAB in industry. Based on these results, K2G30 and the K1G4 (3 days of cultivation), and K2A8, DSM 15973^T^, and DSM 46590 (7 days of cultivation) are candidate strains for surface-microstructured BC production assisted by GAB.

### Characterization of surface-structured bacterial cellulose with guided assembly-based biolithography

**Figure 3:**
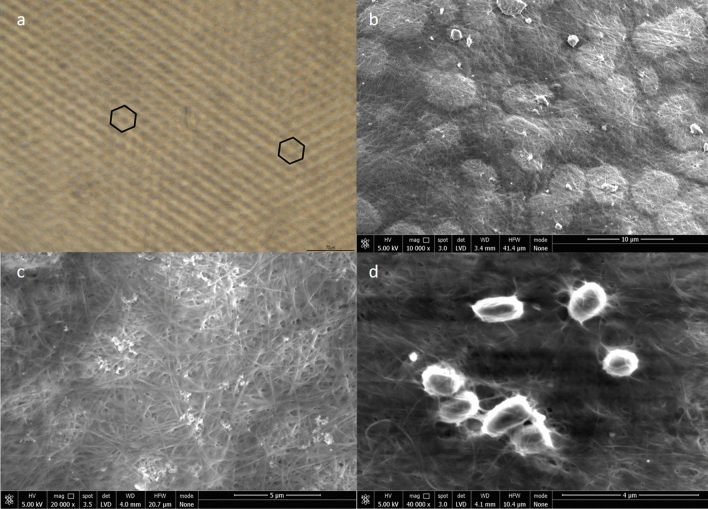
K2A8 strain as representative of the pool of strains. **(a)** Micro-pattern characterization. Hexagonal pattern of the surface-microstructured BC manufactured using biolithography. Hexagonal pattern-formation was observed by BF microscopy at high magnification ×20 using native BC. **(b)** SEM image of surface-microstructured BC. **(c)** SEM image of microstructured BC fibrous network. **(d)** SEM image of K2A8 present among microstructured BC fibrous network.

Since the surface micropattern designed by Hylomorph AG has characteristic dimensions in the sub-micron range (1–10 µm), brightfield microscopy (BF) and scanning electron microscopy (SEM) were used to investigate the presence of the surface micropattern on the pouches. All the 5 candidate strains (K2G30, K1G4, K2A8, DSM 15973^T^ and DSM 46590) showed the hexagonal pattern on the surface using BF (Fig. 3a). In parallel, SEM analysis with high magnification was performed to observe the surface of the matrix and to confirm the fibrous network of BC (Fig. 3b,c). Moreover, to visualize how bacterial cells are dispersed into the BC matrix (Fig. 3d), a K2A8 sample, as representative of the pool of strains, was differently treated for SEM experiment, by turning the BC pouch inside out and reducing the washing steps. Results are consistent with the literature describing AAB of *Komagataeibacter* genus as short rods with an average width of 0.65 µm (ranging from 0.5 to 0.8 µm) and an average length of 2 µm (ranging from 1.0 to 3.00 μm), occurring singly, in pairs or in chain^51^.

## Conclusions

In this work, 25 *Komagataeibacter* strains were tested for producing a BC device suitable for biomedical purposes. The medical device was manufactured in the form of a pouch, synthesized by AAB at the liquid–air interface, after a period of incubation inside a PDMS bioreactor. Among studied strains, K2G30, K1G4, K2A8, DSM 15973^T^ and DSM 46590 produced optimal surface-microstructured BC and they were designated as candidate strains for the purpose of this study. Although a further research step is required to evaluate the biocompatibility, the durability of the device and the reduction of fibrotic tissue, results of this study open new horizons toward applying wild-type AAB strains in the biomedical field.

## Materials and methods

### Materials

HS broth was prepared following the recipe: D-glucose 2% w/v, Yeast extract 1% w/v, Polypeptone 0.5% w/v, Disodium phosphate anhydrous (NaHPO_4_) 0.27% w/v, Citric acid 0.115% w/v)^44^. Whereas the 105 broth was prepared according to DSMZ instructions: D-glucose 10% w/v, calcium carbonate (CaCO_3_) 2% w/v, Yeast extract 1% w/v. Sterilization was conducted in an autoclave at 121 °C for 20 min.

### Bacterial strains and cultivation conditions

AAB strains used in this study were supplied by UMCC (Unimore Microbial Culture Collection, Italy) and DSMZ (Deutsche Sammlung von Mikroorganismen und Zellkulturen GmbH, Braunschweig, Germany) culture collections, respectively (Table 1). All the strains were handled by UMCC culture collection, according to the standard procedures of the Microbial Resource Research Infrastructure—Italian Joint Research Unit (MIRRI-IT)^52^.

DSMZ strains were revitalized according to manufacturer instructions, using 105 broth and UMCC strains on HS broth. After revitalization, a pre-inoculum was performed on 5 mL HS broth. Then an aliquot of inoculum (5% v/v) was transferred in a 100 mL Erlenmeyer flask containing 30 mL HS broth. Incubation was performed at 28 ˚C for 7 days, under static conditions.

### Qualitative and quantitative tests of BC

BC production was qualitatively estimated following the method proposed by Navarro et al., 1999^53^. Pellicle was collected from the broth culture after 7 days of incubation at 28 °C, treated with 4 mL of 5% NaOH and boiled for 2 h. BC production has been confirmed when the pellet did not dissolve after boiling. *K. xylinus* K2G30 was used as a positive control.

Estimation of BC yield was carried out following the method proposed by Hwang et al. 1999^54^. Briefly, native BC from culture broth was collected and washed with distilled water four times with a time-lapse of 15 min and additional washing with 1 M NaOH. Washed BC films were kept at 90 °C for 30 min inside the solution of NaOH 1 M. Finally, BC was rinsed using distilled water four times and then dried at 25 °C until a constant weight was reached. The weighting of the dried BC film was performed using an analytical balance (Gibertini E42S, Milan Italy). The yield of BC was expressed as grams of dried BC per liter (g/L).

### Growth of AAB in 3D bioreactors

3D bioreactors manufactured in PDMS were used as vessels for assessing the development of surface microstructured BC by *Komagataeibacter* genus strains. Cultivation in the bioreactor was carried out with 55 mL of HS broth and 5% v/v inoculum. Bioreactors were covered with sterile gauze on the top and incubated at 28 °C for 7 days. Then, each sample was washed using at first distilled water, then a solution of NaOH 1 M and rinsed with distilled water several times. The last washing in distilled water was conducted in shaking conditions. BC production within the bioreactor was evaluated by the formation of a homogeneous film with a cap at an open liquid–air interface. The sealing of the pouch was removed aseptically and avoiding the damaging of the BC pouch-shaped device. The yield of BC produced was controlled as previously described and expressed in grams of dried BC per liter (g/L).

### Microscopy

The surface microstructure of BC pouches was examined using a field emission scanning electron microscope (NovaNano SEM 450, FEI, USA). Samples were cut (5 × 5 mm^2^) and mounted on a stainless-steel stub with double-sided tape. The analysis was performed in a low vacuum mode (80 kPa) with an acceleration voltage of 10 kV^55^. The surface microstructure pattern of the films was obtained through a BF microscopy Eclipse Ts2 inverted microscope (Nikon, Tokyo, Japan).

### Statistics analysis

The statistical analysis of the data was performed through analysis of variance (ANOVA) using multcompView package implemented in R v 4.0.4^56^. The experiment was performed in 3 replicates. The differences between means were evaluated by Tukey HSD test (p < 0.05). The data were expressed as the mean ± standard deviation (SD).

## Supplementary Information

Supplementary Information.

## Data Availability

All data generated or analysed during this study are included in this published article (and its Supplementary Information file).
