# Supplementary material for: Assessing effectiveness of Komagataeibacter strains for producing surface-microstructured cellulose via guided assembly-based biolithography
Source: Sci Rep. 2021 Sep 29;11:19311. doi: 10.1038/s41598-021-98705-2 (PMC8481549; doi:10.1038/s41598-021-98705-2)
Supplement: Supplementary file 1 — Supplementary Information. [file 41598_2021_98705_MOESM1_ESM.docx]

**Assessing effectiveness of *Komagataeibacter* strains for producing surface-microstructured cellulose via guided assembly-based biolithography**

**Marcello Brugnoli**^1^**, Francesco Robotti**^2^**, Salvatore La China**^1^**, Kavitha Anguluri**^1^**, Hossein Haghighi**^1^**, Simone Bottan**^2^**, Aldo Ferrari**^2,*^**, Maria Gullo**^1,*^

^1^University of Modena and Reggio Emilia, Department of Life Sciences, Reggio Emilia, 42123, Italy

^2^Hylomorph AG, Zurich, Switzerland


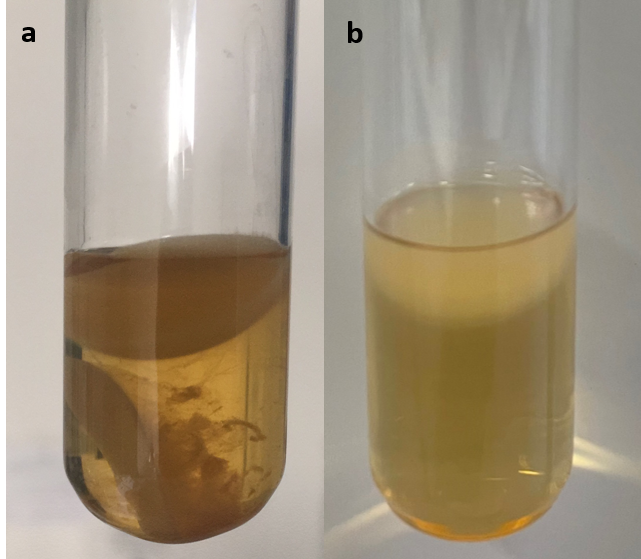


**Supplementary Fig. S1**. Macroscopic structure of native BC in test tubes. (a) Fragmented BC synthetised by DSM 46603 strain. (b) Defined and smooth BC synthetised by K2G30 strain.


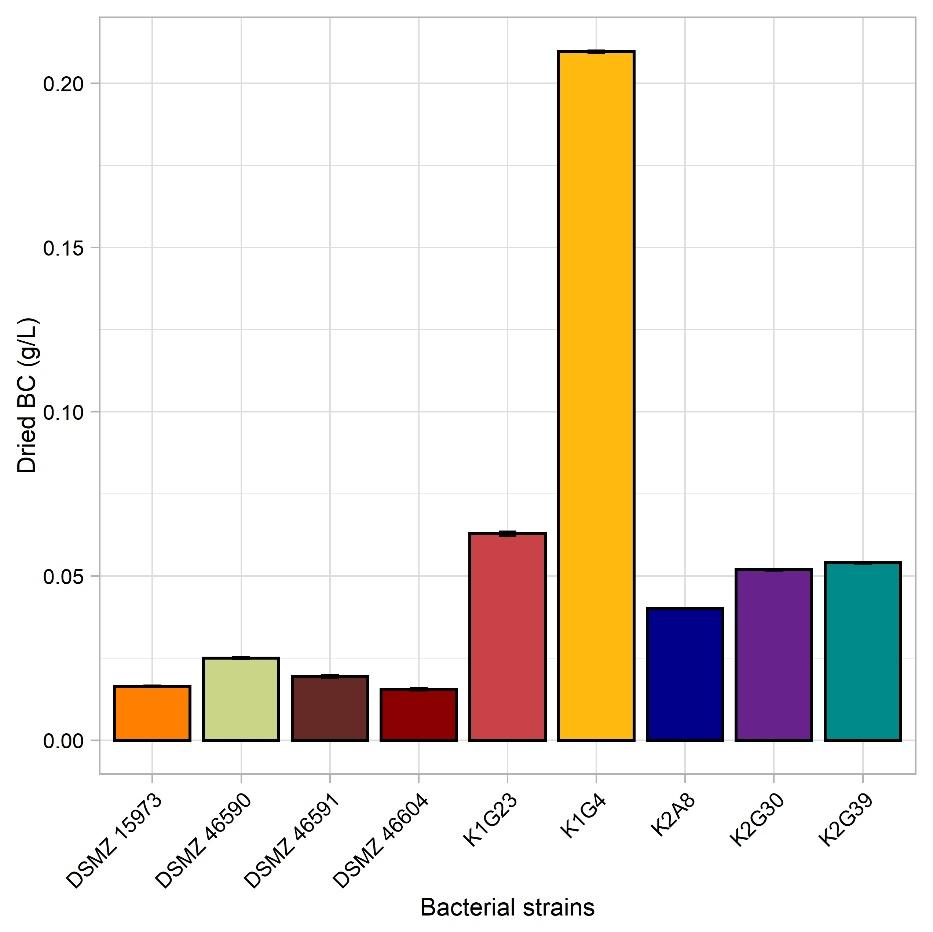


**Supplementary Fig. S2**. BC produced by the 9 strains after incubation at 28 °C for 7 days inside flask. Values are given as mean ± standard deviation (n=3).

**Supplementary Table S1**: Summary table of total native BC production by strains tested in the PDMS bioreactors. BC weighted after cultivation at 28 °C for 7 days inside the bioreactor. Values are given as average ± standard deviation (n =3).

| **Screened strains** | **Native BC (g)** |
| --- | --- |
| **K1G4=UMCC 2947** | 41.4706^ab^ ± 1.4562 |
| **K1G23** | 34.6403^bc^ ± 2.1354 |
| **K2A8** | 31.8669^c^ ± 4.5504 |
| **K2G30=UMCC 2756** | 45.6955^a^ ± 2.2620 |
| **K2G39=UMCC 2970** | 42.7012^ab^ ± 4.3076 |
| **DSM 15973^T^** | 8.0927^d^ ± 1.9003 |
| **DSM 46590** | 8.9974^d^ ± 2.9674 |
| **DSM 46591** | 4.1896^d^ ± 0.9377 |
| **DSM 46604** | 3.1078^d^ ± 0.4243 |
